# Prevalence of Civil Legal Needs and Assistance Among Low-Income US Veterans: Results from the National Veterans Homeless and Poverty Experiences Survey

**DOI:** 10.1007/s10900-026-01604-8

**Published:** 2026-07-22

**Authors:** Melissa R. Cruz, Jack Tsai

**Affiliations:** 1 VA HSR&D Center for Innovations in Quality, Effectiveness, and Safety, Michael E. DeBakey VA Medical Center, 2002 Holcombe Blvd, Houston, TX 77030 USA; 2 Department of Management, Policy, and Community Health, School of Public Health, University of Texas Health Science Center at Houston, Houston, TX USA; 3 U.S. Department of Veterans Affairs, National Center on Homelessness among Veterans, Washington, DC, USA

**Keywords:** Civil legal needs, Legal assistance, Social determinants of health, Low-income, Veterans

## Abstract

Low-income US veterans may experience civil legal needs that intersect with financial, housing, and health-related challenges, yet national data describing these patterns remain limited. This study provides national estimates of civil legal needs and receipt of assistance among veterans with household incomes below 300% of the federal poverty level. Data were drawn from the fifth wave (2025) of the National Veterans Homeless and Other Poverty Experiences (NV-HOPE) study, a nationally representative survey of low-income US veterans (*N* = 1384). Civil legal needs were assessed across seven domains, and receipt of professional assistance was measured among those reporting at least one need. Weighted descriptive statistics and hierarchal logistic regression models examined associations between sociodemographic, clinical, and psychosocial characteristics by reporting a legal need and receiving assistance. Approximately 18% of veterans reported at least one civil legal need, most commonly debt and wills and inheritance. Veterans with legal needs were younger, less likely to be white or married, more likely to have PTSD, and reported higher debt. Among those with a civil legal need, 57% received professional assistance, with notable variation across issue types. Regression analysis showed that factors associated with reporting a legal need differed from those associated with receiving assistance including higher education attainment and naval service. Civil legal needs were reported by a modest proportion of low-income veterans, with distinct characteristics associated with reporting a need and receiving assistance. These findings provide descriptive evidence to inform future research and policy efforts addressing civil legal needs among veterans.

## Introduction

US veterans with low income frequently report civil legal needs, including non-criminal problems related to housing, income security, and family circumstances [1, 2]. Many reflect age-related civil legal needs, as older adults often navigate domains central to later-life stability, including wills and inheritance, Veterans Affairs (VA) benefits, financial security, and housing [1]. Wills and inheritance needs (30.5%) are the most reported, underscoring the relevance of estate planning and end of life decision making in an aging veteran population [1].

Veterans also report challenges related to VA benefits and financial concerns, which may be shaped by fixed incomes, disability status, and the administrative complexity of public benefit systems [1, 2]. Among low-income veterans with histories of homelessness, difficulties accessing or maintaining benefits are documented [3, 4], and these challenges appear alongside housing- and benefits-related legal issues reported across homeless service settings [2, 4, 5] where more than 90% of sites note at least one civil legal need [2].

Despite the frequency of these age-linked legal needs, routine screening and integrated legal services remain limited with health and social service systems [2]. Recent federal policy developments provide additional context. The Legal Services for Homeless Veterans and Veterans At-Risk for Homelessness (LSV-H) program, authorized under Public Law 116–315, was established to strengthen community-based delivery of legal services for veterans [6], informed by national estimates of substantial unmet need and the complexity of issues such eviction related concerns and disability benefits navigation [7, 8]. Through grants to nonprofit organizations and law school clinics, LSV-H prioritizes services for women veterans, rural and tribal communities, and veterans experiencing or at risk for homelessness [7].

Existing studies provide little information on how sociodemographic, clinical, and psychosocial characteristics relate to these outcomes in later life [1]. To address this gap, the present study uses a nationally representative sample of veterans to examine factors associated with (1) reporting at least one civil legal need in the past year and (2) receiving assistance from a professional. This analysis aims to clarify which subgroups of veterans report legal needs and which are more likely to receive help, with the goal of informing future efforts to support veterans facing civil legal challenges.

## Methods

The National Homeless and Other Poverty Experiences (NV-HOPE) study is a series of nationally representative surveys funded by the US Department of Veterans Affairs that examines the determinants of health, housing stability and other psychosocial outcomes among low-income veterans [3, 9]. The NV-HOPE study was developed through a contract with Ipsos, which administered surveys using its KnowledgePanel. KnowledgePanel is designed to be representative of the US population, including individuals who are typically underrepresented in online research, such as households without internet access and Spanish-language-dominant respondents. Eligibility criteria for NV-Hope included being 18 years or older, having served on active duty in the US Armed Forces, and living in a household below 300% of the federal poverty level in 2021. The baseline survey was fielded from October 28 to December 3, 2021. A total of 2,057 individuals were invited to participate, and 1,577 (76.7%) completed the survey. Of those who completed the survey, 1004 met all inclusion criteria and were included in the NV-HOPE analytic sample. More details about the sampling and poststratification weights have been described elsewhere [3, 9]. This study focused on the NV-HOPE wave 5 data collected between November 4 and December 9, 2025. The Wave 5 data included both a follow-up survey to the original cohort (*N* = 812) and a newly recruited cohort during this wave (*N* = 572), yielding a combined analytical sample of 1,384 low-income veterans with household incomes below 300% of the federal poverty level.

### Measures

#### Sociodemographic Characteristics

Sociodemographic variables included age, gender, race/ethnicity, education level, marital status, annual household income, debt level, and military service background, employment status, and current housing situation.

#### Civil Legal Needs

Civil legal needs were assessed by asking the question, ‘Now we want to ask you about non-criminal problems. In the past year, did you need any help with any of the following?’ and responded yes/no to the following: ‘housing,’ ‘child support issues,’ ‘divorce,’ ‘wills and inheritance,’ ‘military discharge upgrade,’ ‘debt,’ and ‘employment problems.’ Any veterans who endorsed any item were classified as having ‘needed legal help.’ A summary indicator was created to identify veterans who endorsed multiple legal problem domains (≥ 2 items).

#### Received Professional Help

Participants who reported a legal need were asked, ‘Were you able to get help from a legal professional for any of the following issues you mentioned you needed help for in the past year?’ and responded yes/no.to the following: ‘housing,’ ‘child support issues,’ ‘divorce,’ ‘wills and inheritance,’ ‘military discharge upgrade,’ ‘debt,’ and ‘employment problems.’ Any veterans who endorsed any item were classified as having ‘received help.’ A summary indicator was created to identify veterans who endorsed multiple legal problem domains (≥ 2 items).

#### Clinical Status

Clinical status was assessed using a checklist of nine lifetime psychiatric diagnoses. Participants were asked, ‘In the past year, has a doctor or nurse informed you that you have any of the following conditions: schizophrenia/schizoaffective disorder (SD), posttraumatic stress disorder (PTSD), alcohol use disorder (AUD), bipolar disorder (BD), generalized anxiety disorder (GAD), major depressive disorder (DD), drug use disorder (DUD), traumatic brain injury, other mental health disorder.’ Item responses AUD and DUD were collapsed into any substance use disorder (ASUD); and SD and BD collapsed into any severe mental illness (ASMI). Any lifetime suicidal attempt was coded based on responses to ’How many times have you tried to kill yourself?’

#### Psychosocial Status

Current housing status was assessed with nine response options collapsed into five categories: own home/apartment, subsidized housing (e.g., Sect.  8), nursing home/long-term care facility (LTCF), transient housing situation (e.g., staying with someone, hotel/motel, transitional housing), and homeless (e.g., shelter, vehicle, outdoors). Employment status was assessed with six response options collapsed into four categories: employed (full- or part time), unemployed, self-employed/other, and retired/disabled. Personal debt was assessed using 15 response options collapsed into six categories: none, <$5,000; $5,000-$15,000; $15,0001–65,000; $65,001-105,000; >$105,000.

### Data Analysis

Descriptive analyses were conducted to examine the civil legal needs of veterans and receipt of professional assistance. Bivariate comparisons between veterans with and without legal needs were conducted using t-tests and Chi-squared tests. Levene’s test for equality of variances was used and corrections to t-tests were applied when appropriate. Hierarchical logistic regression analyses were conducted to identify characteristics independently associated with (1) needing non-criminal legal help and (2) receiving professional assistance. Sociodemographic characteristics were entered in Block 1, clinical characteristics were entered in Block 2, and psychosocial characteristics in Block 3. Odds ratios (OR) were calculated with 95% confidence intervals (Cis), and Nagelkerke R^2^ was used to estimate the variance explained. Listwise deletion was applied to all analyses.

## Results

Across the full sample (*n* = 1,384), 250 (weighted 18%) reported at least one civil legal need in the past year. Veterans with legal needs differed significantly from the broader study population on sociodemographic, economic, housing, and clinical characteristics. As shown in Table 1, veterans needing help were younger (64.9 vs. 68.4 years; t(1383) = 4.61, *p*<0.001), less likely to be White, non-Hispanic (39.8% vs. 51.8%; $\:{\chi\:}^{2}$(3) = 22.48, *p*<0.0001) and less likely to be married (19.8% vs.27.9%, $\:{\chi\:}^{2}$(1) = 15.07, *p*<0.0001). Lower household incomes and higher levels of personal debt were also more common among those needing civil legal assistance, with debt showing a particularly strong association ($\:{\chi\:}^{2}$(5) = 50.89, *p*<0.0001). Veterans needing civil legal assistance were less likely to live in their own home or apartment (69.6% vs. 83.4%, $\:{\chi\:}^{2}$(4) = 22.52, *p*<0.0001). Clinically, they had higher rates of PTSD (18.0% vs.7.8%), major depressive disorder (12.4% vs.5.3%), generalized anxiety disorder (6.4% vs. 2.2%), serious mental illness (4.8% vs. 1.7%), and lifetime suicide attempt (4.6% vs.2.3%), with all comparisons significant at *p*<0.001.

**Table 1:** Characteristics of Veterans Who Needed Civil Legal Assistance

	Needed Civil Legal Assistance (*N* = 250)	Study population (*N* = 1384)	Test of difference
Age	64.9 (13.8)	68.4 (13.8)	t (1383) = 4.61***
Gender			
Male	215 (75.4%)	1188 (75.2%)	$$\:{\chi\:}^{2}$$(1) = 0.003
Female	35 (24.6%)	196 (24.8%)	
Race/ethnicity			
White, non-Hispanic	162 (39.8%)	1046 (51.8%)	$$\:{\chi\:}^{2}$$(3) = 22.48***
Black, non-Hispanic	44 (21.6%)	155 (15.4%)	
Other, non-Hispanic	19 (14.0%)	69 (10.3%)	
Hispanic	25 (24.6%)	114 (22.6%)	
Education			
Less than high school	6 (0.8%)	17 (0.4%)	$$\:{\chi\:}^{2}$$(3) = 3.46
High school	48 (12.5%)	279 (13.1%)	
Some college	120 (47.0%)	682 (48.2%)	
Bachelor’s degree or higher	76 (39.7%)	406 (38.3%)	
Married	113 (19.8%)	778 (27.9%)	$$\:{\chi\:}^{2}$$(1) = 15.07***
Annual household income			
<$10,000	20 (2.5%)	49 (1.0%)	$$\:{\chi\:}^{2}$$(6) = 20.21**
$10,000–24,999	47 (11.8%)	225 (9.6%)	
$25,000–49,999	98 (36.9%)	545 (35.0%)	
$50,000–74,999	45 (22.6%)	373 (31.9%)	
$75,000–99,999	32 (20.1%)	120 (12.8%)	
$100,000-149,999	8 (6.0%)	58 (7.4%)	
$150,000 or more	0 (0%)	14 (2.1%)	
Personal Debt			
None	31 (3.8%)	359 (9.8%)	$$\:{\chi\:}^{2}$$(5) = 50.89***
<$5000	39 (9.6%)	275 (15.1%)	
$5,001–15,000	35 (12.9%)	147 (12.1%)	
$15,001–65,000	65 (32.0%)	232 (25.4%)	
$36,001-105,000	15 (9.2%)	76 (10.4%)	
>$105,000	44 (32.5%)	166 (27.3%)	
Service branch			
Army	100 (15.1%)	517 (13.7%)	$$\:{\chi\:}^{2}$$(1) = 1.51
Air Force	39 (11.7%)	255 (13.6%)	$$\:{\chi\:}^{2}$$(1) = 2.96
Marine Corps	25 (11.3%)	105 (8.4%)	$$\:{\chi\:}^{2}$$(1) = 1.91
Navy	52 (31.3%)	304 (32.3%)	$$\:{\chi\:}^{2}$$(1) = 0.00
Coast guard	1 (0.8%)	14 (1.9%)	$$\:{\chi\:}^{2}$$(1) = 0.48
National guard/reserves	33 (29.8%)	189 (30.1%)	$$\:{\chi\:}^{2}$$(1) = 0.06
Employment Status			
Employed	59 (9.8%)	260 (7.3%)	$$\:{\chi\:}^{2}$$(3) = 10.47*
Unemployed	11 (3.7%)	37 (2.1%)	
Retired	167 (83.2%)	1028 (86.7%)	
Self-employed/other	13 (3.3%)	59 (3.9%)	
Current housing situation			
In own home/apartment	215 (69.6%)	1283 (83.4%)	$$\:{\chi\:}^{2}$$(4) = 22.52***
In subsidized housing	19 (12.3%)	55 (7.1%)	
In nursing home/LTCF	1 (1.0%)	5 (1.0%)	
Transient housing	12 (15.5%)	29 (7.5%)	
Homeless	1 (1.6%)	3 (1.0%)	
Lifetime psychiatric diagnoses			
PTSD	45 (18.0%)	108 (7.8%)	$$\:{\chi\:}^{2}$$(1) = 46.53***
Major depressive disorder	31 (12.4%)	73 (5.3%)	$$\:{\chi\:}^{2}$$(1) = 33.78***
Generalized anxiety disorder	30 (12.0%)	89 (6.4%)	$$\:{\chi\:}^{2}$$(1) = 16.00***
Any substance use disorder	16 (6.4%)	30 (2.2%)	$$\:{\chi\:}^{2}$$(1) = 25.47***
Any serious mental illness	12 (4.8%)	24 (1.7%)	$$\:{\chi\:}^{2}$$(1) = 16.62***
Positive screen for any lifetime suicidal attempt	22 (4.6%)	61 (2.3%)	$$\:{\chi\:}^{2}$$(1) = 14.41***

LTCF: Longterm care facility; PTSD: Posttraumatic stress disorder; *p < 0.05; **p < 0.01; ***p < 0.001

Among veterans reporting a legal need, the most common issues were debt (53.6%), wills and inheritance (37.6%), housing (16.0%), and employment (16.4%) (Table 2). Approximately 22.8% reported civil legal needs across multiple domains, while three in four veterans (77.2%) reported legal problems confined to a single domain $\:{\chi\:}^{2}$(1) = 66.12, *p* < 0.0001). Overall, 57% (weighted; *N* = 142 veterans) received professional assistance. Veterans with wills and inheritance issues were significantly more likely to obtain assistance (50% vs. 37.6%, $\:{\chi\:}^{2}$(1) = 26.71, *p*<0.0001), whereas those with debt-related problems were less likely to receive assistance (38.0% vs. 53.6%, $\:{\chi\:}^{2}$(1) = 4.67, *p*<0.031).

**Table 2:** Civil Legal Problems Experienced and Receipt of Professional Help Among Veterans

Legal Problem Domain	Needed Legal Help (*N* = 250)	Received Help (*N* = 142)	Test of difference
Multiple domains (≥ 2) ª	57 (22.8%)	21 (14.8%)	$\:{\chi\:}^{2}$(1) = 66.12***
Single domain	193 (77.2%)	121 (85.2%)	
Housing Issues	40 (16.0%)	16 (11.3%)	$\:{\chi\:}^{2}$(1) = 1.68
Child support / Family Law	15 (6.0%)	6 (4.2%)	$\:{\chi\:}^{2}$(1) = 1.84
Divorce	20 (8.0%)	12 (8.5%)	$\:{\chi\:}^{2}$(1) = 0.59
Wills and inheritance	94 (37.6%)	71 (50.0%)	$\:{\chi\:}^{2}$(1) = 26.71***
Military Discharge	20 (8.0%)	3 (2.1%)	$\:{\chi\:}^{2}$(1) = 1.20
Debt	134 (53.6%)	54 (38.0%)	$\:{\chi\:}^{2}$(1) = 4.67^*^
Employment Problems	41 (16.4%)	14 (9.9%)	$\:{\chi\:}^{2}$(1) = 0.62

^a^ Reflects the number and weighted percentage of veterans who endorsed more than one legal domain. *p < 0.05; **p < 0.01; ***p < 0.001

Hierarchal logistic regression models identified several factors associated with reporting a civil legal need (Table 3). Lower odds of civil legal need were observed among White veterans (OR = 0.64, 95% CI = 0.43–0.94, *p* = 0.024) and those who were married (OR = 0.67, 95% CI = 0.48–0.95, *p* = 0.026). Sociodemographic characteristics together explained 6% of the variance in needing non-criminal legal help. Clinical characteristics, in Block 2, explained an additional 9% of the variance. PTSD (OR = 2.31, 95% CI = 1.34–3.99, *p* = 0.002) and ASUD (OR = 2.12, 95% CI = 1.18–3.82, *p* = 0.012) were associated with more than twice the likelihood of reporting a non-criminal legal need. Living in one’s own home was protective (OR = 0.55, 95% CI = 0.32–0.96, *p* = 0.034), whereas higher levels of personal debt were a strong predictor of legal need (OR = 1.33, 95% CI = 1.21–1.46, *p* < 0.0001). The final model explained 14% of the variance in legal need.

**Table 3:** Logistic Regression of Characteristics Associated with Needed Help for Civil Legal Problems Among Veterans

	Odds Ratio	95% Confidence Interval
**Sociodemographics**		
Age	0.99	0.98-1.00
Male	0.75	0.48–1.19
White (ref. non-White)	0.64	0.43–0.94*
Hispanic (ref. non-Hispanic)	0.93	0.51–1.71
Education level	1.13	0.95–1.35
Married	0.67	0.48–0.95*
Income Level	0.90	0.78–1.04
Service in Navy (ref. other branches)	1.28	0.89–1.84
*Nagelkerke R* ^*2*^	0.06	
**Clinical Status**		
PTSD	2.31	1.34–3.99**
Depressive disorder	1.53	0.75–3.10
General Anxiety disorder	0.83	0.42–1.65
Any substance use disorder	2.25	0.29–5.68
Any serious mental illness	1.32	0.49–3.54
Positive screen for any lifetime suicidal attempt	1.92	0.42–8.87
*Nagelkerke R* ^*2*^	0.09	
**Psychosocial status**		
In own housing (Ref. other housing)	0.55	0.32–0.96*
Employed (Ref. not employed)	0.95	0.60–1.48
Personal debt	1.33	1.21–1.46***
*Nagelkerke R* ^*2*^	0.14	

PTSD: Posttraumatic stress disorder; *p < 0.05; **p < 0.01; ***p < 0.001

Among veterans with civil legal need, predictors of receiving professional assistance differed (Table 4). Higher educational attainment increased the likelihood of receiving professional assistance (OR = 1.48, 95% CI = 1.05–2.09, *p* = 0.025). Veterans who served in the Navy had more than twice the odds of receiving professional assistance for their non-criminal legal needs compared to veterans from other branches (OR = 2.33, 95% CI = 1.12–4.85, *p* = 0.023). Sociodemographic characteristics together explained 14% of the variance in receiving professional assistance and the addition of clinical characteristics explained an additional 18%. Clinical and psychosocial characteristics were not significant predictors of receiving professional assistance. The final model accounted for 19% of the variance in receiving professional assistance.

**Table 4:** Logistic Regression of Characteristics Associated with Received Help for Civil Problems Among Veterans

	Odds Ratio	95% Confidence Interval
**Sociodemographics**		
Age	1.02	1.00-1.05
Male	1.45	0.60–3.47
White (ref. non-White)	1.36	0.67–2.78
Hispanic (ref. non-Hispanic)	0.82	0.27–2.45
Education level	1.48	1.05–2.09^*^
Married	1.30	0.68–2.48
Income Level	1.16	0.90–1.49
Service in Navy (ref. other branches)	2.33	1.12–4.85^*^
*Nagelkerke R* ^*2*^	0.14	
**Clinical Status**		
PTSD	2.45	0.94–6.39
Depressive disorder	0.39	0.13–1.20
General Anxiety disorder	0.65	0.21–2.03
Any substance use disorder	1.60	0.31–8.19
Any serious mental illness	0.50	0.12–2.11
Positive screen for any lifetime suicidal attempt	1.46	0.09–24.24
*Nagelkerke R* ^*2*^	0.18	
**Psychosocial status**		
In own housing (Ref. other housing)	0.93	0.35–2.47
Employed (Ref. not employed)	0.64	0.29–1.44
Personal debt	0.93	0.77–1.12
*Nagelkerke R* ^*2*^	0.19	

PTSD: Posttraumatic stress disorder; *p < 0.05; **p < 0.01; ***p < 0.001

## Discussion

In this nationally representative sample of low-income older veterans, a modest but meaningful proportion reported at least one civil legal need, most commonly involving debt, wills and inheritance, housing, and employment. Legal needs varied across sociodemographic and clinical characteristics, and only a subset of veterans with legal need reported professional assistance. Characteristics associated with reporting a legal need differed from those associated with receiving assistance, suggesting that identification of legal issues and engagement with services may be shaped by distinct processes. These findings contribute descriptive evidence on how civil legal needs and service use are distributed across veteran subgroups.

Patterns observed in this study align with prior work, documenting higher civil legal need among veteran who are younger, less likely to be white or married, and more likely to experience financial strain, housing stability, and psychiatric conditions [1]. Although prevalence of legal needs in this study (18% weighted) was lower than previously reported estimates (53.9% weighted), differences in sampling frames and potential response bias, such as greater participation among veterans with unmet needs, may help contextualize this variation [1]. Similar patterns have been described in studies of veterans engaged with medical-legal partnerships, which report concentrations of housing, benefits, and family-related issues among veterans with sociodemographic disadvantage and mental health conditions [2, 4, 5]. Nonetheless, the findings are consistent with other studies pointing to connections between civil legal issues and mental health [10, 11] as well as certain sociodemographic subgroups that may be more likely to have civil legal needs in the general population [12–14].

Among veterans who had civil legal issues, this study found 57% reported receiving professional assistance for their civil legal issue suggesting more than half of veterans do receive legal assistance. This estimate is a bit higher than reported in a previous study that found 42.8% of veterans who reporting needing civil legal aid received it [1]. Also different from that study, this study found that higher educational attainment and Navy service were associated with receiving assistance, while clinical and psychosocial characteristics were not associated with receipt of civil legal aid. Both studies indicate substantial unmet civil legal needs with differences across legal domains, including higher assistance for wills and inheritance issues and lower assistance for debt-related concerns [1]. Earlier studies of medical-legal partnerships, which included only veterans who accessed services, focused primarily on issues addressed rather than the unmet needs or characteristics linked to receiving assistance [2, 4, 5].

These findings correspond with national efforts to expand access to legal support for veterans. The VA’s LSV-H program aims to strengthen community-based legal service delivery in response to documented unmet need [6]. Although the present study does not evaluate LSV-H, its descriptive patterns may help identify low-income veterans who report legal needs and those who appear less likely to receive assistance.

Future research may clarify structural, informational, and logistical factors that shape whether veterans with civil legal needs obtain assistance. Qualitative interviews with veterans who reported a legal need but did not receive help provide insight into how legal issues are understood and what influenced decisions to seek assistance. As VA and community partners continue to develop legal service programs, integrating outreach and navigation support into existing health and social service settings may help older veterans more effectively identify and access available resources.

Several study limitations are important to note. The cross-sectional design limits temporal interpretation of survey-based measures, and all measures relied on self-report. Legal needs were captured through brief checklist items, which may not fully reflect the complexity of veterans’ civil legal experiences. The survey did not include information on the type or outcomes of legal assistance, limiting the ability to describe how support aligns with unmet legal needs. Strengths of the study include the use of a nationally representative veteran sample, standardized survey methods, and detailed assessment of civil legal needs and assistance received.

## Conclusion

The national study describes civil legal needs among older, low-income veterans and shows that characteristics associated with receiving assistance differ from those associated with reporting a need. These distinctions help identify where gaps in access may occur and which subgroups of veterans may be less likely to obtain support. The findings may also be informative for ongoing national efforts to expand legal service delivery for veterans, including programs such as the LSV-H program. Future work could further examine structural, informational, and logistical factors that shape whether veterans with civil legal needs obtain assistance. These findings may contribute to the growing knowledge base informing practice, research and policy development related to legal service programs for veterans.

## Data Availability

The dataset generated and analyzed during the NV-HOPE study are not publicly available, but de-identified data may be made available from the corresponding author upon reasonable request.
